# Integrated metabolomics and transcriptomics reveal the potential of hydroxy-alpha-sanshool in alleviating insulin resistance

**DOI:** 10.1186/s10020-025-01129-1

**Published:** 2025-02-21

**Authors:** Yuping Zhu, Pan Yang, Tingyuan Ren, Zhuqi Chen, Huanhuan Tian, Mingfen Wang, Chunlin Zhang

**Affiliations:** 1 School of Basic Medicine, Guizhou Medical University, Guiyang, 550025 China; 2 College of Brewing and Food Engineering, Guizhou University, Guiyang, 550025 China; 3 School of Clinical Medicine, Guizhou Medical University, Guiyang, 550025 China

**Keywords:** Hydroxy-alpha-sanshool, Insulin resistance, Transcriptomics, Metabolomics

## Abstract

Hydroxy-alpha-sanshool (HAS) has attracted attention because of its various biological activities, such as hypoglycemic, hypolipidemic, and antioxidant activities. In this study, we investigated the effects of HAS on insulin resistance (IR) and its mechanism. HAS reduced fasting blood glucose (FBG), promoted insulin (INS) secretion, significantly decreased levels of interleukin (IL)-1, IL-6, tumor necrosis factor (TNF)-α and monocyte chemoattractant protein-1 (MCP-1), and increased the IL-2 level in serum of IR model mice. HAS regulated the mRNA levels of protein kinase B (Akt), B-cell lymphoma extra-large (Bcl-xL), stearoyl-CoA desaturase-1 (SCD1), nuclear factor kappa B (NF-κB), and eukaryotic translation initiation factor 4E (eIF4E). Additionally, differentially abundant metabolites in IR model mice treated with HAS were involved in these signaling pathways including prion disease, choline metabolism in cancer, regulation of lipolysis in adipocytes and the pentose phosphate pathway and positively regulated betaine abundance. In conclusion, HAS activated the phosphatidylinositol-3 kinase (PI3K)/Akt insulin and NF-κB signaling pathways to maintain glucose homeostasis and regulate IR.

**Supplementary Information:**

The online version contains supplementary material available at 10.1186/s10020-025-01129-1.

## Introduction

Diabetes mellitus (DM) is one of the four leading noncommunicable diseases worldwide and one of the three leading causes of death (Saeedi et al. 2020). The common subtypes of DM include type 1 DM (T1DM) and type 2 DM (T2DM). T2DM is the most common type of DM, accounting for approximately 90–95% of DM cases. Insulin resistance (IR) is one of the main pathogenic mechanisms of T2DM, and obesity is an important factor leading to IR. During long-term obesity, the substantial deposition of organ and peripheral fat reduces target organ sensitivity to insulin (INS) (Barazzoni et al. 2018). Studies have shown that obesity can cause metabolic stress, increase reactive oxygen species (ROS), activate p53, and increase the production of proinflammatory factors, causing adipose tissue inflammation, cell senescence and IR (Minamino et al. 2009; Shimizu et al. 2013). The signaling pathways involved in the regulation of INS metabolism are very important for maintaining whole-body homeostasis. Therefore, IR has long been the focus of multidisciplinary research in the medical field. Currently, the treatments for IR mainly include changing daily lifestyles (such as changing diet, increasing exercise, and losing weight) and clinical drug intervention (such as the use of INS sensitizers).

*Zanthoxylum bungeanum* is the fruit of the genus *Zanthxylum L.* in the *Rutaceae* family and is a traditional spice and Chinese medicine. It has been shown that the amides in the peel of *Z. bungeanum* are composed mainly of hydroxy-α-sanshool (HAS), hydroxy-β-sanshool (HBS) and hydroxy-γ-sanshool (HRS), with HAS accounting for more than 95% of the components; these molecules are long-chain unsaturated fatty acid amides (Xu et al. 2022a, b). Structure-activity studies have revealed that HAS, HBS and HRS are associated with numbness, with HAS showing the strongest effect. The possible mechanisms of action are related to the activation of TRPV1 and TRPA1 channels (Koo et al. 2007) and the blockade of a two-pore domain potassium channel (K2P) (Watanabe and Terada 2015) by HAS. Wang Li et al. (Wang et al. 2019) showed that HAS increased the levels of reduced glutathione and superoxide dismutase (SOD) in the livers of hyperlipidemic mice and reduced the malondialdehyde (MDA) level, confirming that HAS can reduce oxidative stress in the liver tissues of mice under high-fat stress. Li et al. (2020) reported that HAS can activate the phosphatidylinositol-3 kinase (PI3K)/Akt signaling pathway to inhibit H_2_O_2_-induced cell apoptosis. Our previous studies revealed that HAS can activate the mammalian target of rapamycin (mTOR) and adenosine monophosphate (AMP)-activated protein kinase (AMPK) signaling pathways to ameliorate glucose, lipid and protein metabolic disorders in diabetic rats (You et al. 2015; Ren et al. 2017a, b); activate the Shh/Ptch1/Smo signaling pathway to alleviate cognitive dysfunction in Alzheimer’s disease model mice (Li et al. 2024a, b); and improve intestinal health in IR model mice (Xu et al. 2022a, b). However, the effect and mechanism of HAS on IR induced by a high-fat diet have not been reported.

In this study, in vivo animal experiments and transcriptomic and metabolomic analyses were performed to elucidate the regulatory mechanism of HAS on energy–lipid–inflammation interactions in the development of IR in a high-fat diet-induced IR mouse model. The results provide new ideas and targets for the application of HAS and prevention of IR via natural active dietary ingredients.

## Materials and methods

### Experimental materials

Ninety specific-pathogen-free (SPF)-grade Kunming male mice weighing 18–20 g were purchased from Liaoning Changsheng Biotechnology Co., Ltd. [production license number: SCXK (Liao) 2015–0001]. HAS was procured from Shanghai Yuanye Biotechnology Co., Ltd. [CAS No. 83883-10-7 and article number B26430 (HPLC ≥ 98%)]. Streptozotocin (STZ) was obtained from Sigma‒Aldrich (St. Louis, MO, USA). The fluorescence reagents and consumables were obtained from Bio-Rad. Triglyceride (TG), total cholesterol (TC), high-density lipoprotein cholesterol (HDL-C), glycosylated serum protein (GSP), low-density lipoprotein cholesterol (LDL-C), glycosylated hemoglobin (GHb), and INS levels were determined via enzyme-linked immunosorbent assay (ELISA) kits (Jiancheng, Nanjing, China). Antibodies against ACLY, ACC, FASN, stearoyl-CoA desaturase-1 (SCD1), GCLM, GSR, HO1, Nrf2, CHOP, IRE, eIF2α and Bip were purchased from Abcam.

### Animal husbandry and sample collection

Ninety Kunming SPF male mice weighing 20–22 g were adaptively fed for 7 days in metabolic cages. The mice were randomly divided into a blank group (BG, *n* = 12) and a high-fat and high-glucose diet group (*n* = 78) according to body weight. The mice in the BG were fed basal feed, and the mice in the high-fat and high-glucose diet groups were fed a high-fat and high-glucose diet. The mice in both groups had free access to water and food. After 8 weeks of feeding, the animals were fasted for 12 h. Citrate buffer was injected intraperitoneally into the mice in the BG, and 60 mg/kg bw STZ was injected intraperitoneally into the mice in the high-fat and high-glucose diet group. During the modeling process, the mice in each group had free access to water and food. On day 7 after modeling, after fasting for 12 h, blood was collected from the tip of the tail, and fasting blood glucose (FBG) was measured with a glucometer. When the FBG was greater than 11.10 mmol/L (Xu et al. 2022a, b; Begorre et al. 2017), modeling was considered successful. Sixty model mice were randomly divided into the model group (MG), metformin hydrochloride group [100 mg/ (kg·bw) (PC)], high-dose HAS group [12 mg/ (kg.d·bw) (HG)], medium-dose HAS group [8 mg/ (kg.d·bw) (DG)], and low-dose HAS group [4 mg/ (kg.d·bw) (LG)], with 12 animals in each group. The body weights of the mice were recorded every week, and the doses were adjusted accordingly. Blood was collected from the tip of the tail on days 0, 14, and 28. FBG was measured with a glucometer. The high-fat and high-glucose diet and dose of HSA used for gavage were selected according to previous study results (Wang et al. 2019; You et al. 2015; Ren et al. 2017a, b; Li et al. 2024a, b).

On day 29, after a 12 h fast, retroorbital blood was collected from the mice and placed in centrifuge tubes. The samples were immediately centrifuged at 4000 r/min for 0.5 h and then at 4 °C for 15 min. The serum layer was stored at -80 °C. The mice were quickly dissected on ice, and the liver and kidneys were removed. The liver and kidneys were rinsed with normal saline to remove blood and then weighed. The liver and kidneys were placed into a centrifuge tube, snap frozen in liquid nitrogen, and then transferred to a -80 °C freezer for subsequent tests.

### Measurement of physiological and biochemical indicators

TC, TG, HDL-C, LDL-C, catalase (CAT), MDA, SOD, GSP, GHb, INS, interleukin-1 (IL-6), interleukin-2 (IL-2), interleukin-6 (IL-6), tumor necrosis factor-α (TNF-α), monocyte chemoattractant protein 1 (MCP-1), adenosine triphosphate (ATP), adenosine diphosphate (ADP), and AMP levels were determined according to the instructions of the kits produced by Nanjing Jiancheng Bioengineering Institute.

### Oral glucose tolerance test

1 day before the end of the experiment, after fasting for 12 h, mice were given an oral 2.0 g/kg.bw glucose solution, and blood was collected from the tip of the tail at 0, 0.5, 1 and 2 h after oral glucose, and blood glucose levels were measured by glucose meter. These data points are used to plot a curve, and calculate the area under the curve (AUC). The AUC calculation formula is as follows:

$$\:\text{AUC}/ (\text{hmmol}/\text{L})=\frac{\text{0.5A+B+C+0.5D}}{2}$$

A, B, C and D are blood glucose values at 0, 0.5, 1 and 2 h after oral glucose.

### Pathological observation of liver tissues

Liver tissue samples were collected, fixed in 4% paraformaldehyde, embedded in paraffin, dehydrated, sectioned, and stained with hematoxylin‒eosin (HE). Pathological changes in the liver tissue were observed under a light microscope. Liver tissues were placed in optimal cutting temperature compound, and frozen sections were placed in oil red O staining solution and mounted with glycerol gelatin. The formation of lipid droplets in the liver tissue was observed under a light microscope.

### Transcriptomics and metabolomics

#### Transcriptome sequencing

Liver tissues from 12 mice in each group were pooled into three parallel samples, which included liver tissues from mice #1, 3, 7, and 11; #2, #4, #8, and #10; and #5, #6, #9, and #12. The transcriptome sequencing of the liver tissue samples in this study was completed by Beijing Biomarker Technologies Co., Ltd. RNA was extracted via a TRIzol kit. Poly (A) mRNA was purified from total RNA via mRNA capture beads, and a reverse transcription primer was designed to anneal to the poly (A) tail of the mRNA. First-strand cDNA was synthesized via reverse transcriptase (RT); polymerase chain reaction (PCR) amplification primers were used to amplify double-stranded cDNA (ds-cDNA), and NEBNext FFPE DNA Repair Mix and the NEBEext Ultra II End Repair/dA-Tailing Module were used for end repair and adenine addition to nucleic acid fragments. A SQK-LSK109 kit was used to complete the ligation of the sequencing adapters, the libraries were configured, and a PromethION48 sequencer was used for sequencing, with 10 biological replicates per group. After sequencing, the raw data were filtered to obtain total high-quality data (clean data) for subsequent bioinformatics analysis. A fold change (FC) ≥ 1.5 and *P* < 0.05 were used as screening criteria, and the obtained differentially expressed genes (DEGs) were subjected to Gene Ontology (GO) and Kyoto Encyclopedia of Genes and Genomes (KEGG) analyses.

#### Analysis of mRNA expression

Complementary DNA (cDNA) was obtained via mRNA extraction and reverse transcription via Qiagen kits. The RNA concentration and optical density (OD) at 260 nm/OD280 nm were measured with an ultraviolet (UV) spectrophotometer (NanoDrop 1000). The expression level of each target gene was measured via real-time PCR (qRT‒PCR). The relative expression level of each target gene was calculated via the Ct^− 2ΔΔt^ method, with β-actin used as an internal reference (Chang et al. 2010). For the sequences of the primers and amplified fragments, see Supplementary Table 1.

#### Untargeted metabolomics (UPLC/HRMS)

For UPLC/HRMS, liver tissue processing was the same as that used for transcriptomic sequencing. UPLC/HRMS was performed by Beijing Biomarker Technologies Co., Ltd. The chromatographic conditions were as follows: ACQUITY UPLC^®^ HSS T3 1.8 μm (2.1 × 150 mm) column (Waters, Milford, MA, USA); autosampler temperature, 8 °C; gradient elution, flow rate of 0.25 mL/min; column temperature, 40 ℃; injection volume, 2 µL; mobile phase (positive ion mode), 0.1% formic acid in water (C)-0.1% formic acid in acetonitrile (D); and mobile phase (negative ion mode), 5 mM ammonium formate solution (A)-acetonitrile (B). The gradient elution program was as follows: 0–1 min, 2% B/D; 1–9 min, 2–50% B/D; 9–12 min, 50–98% B/D; 12–13.5 min, 98% B/D; 13.5–14 min, 98–2% B/D; and 14–20 min, 2% D-positive mode (14–17 min, 2% B-negative mode). The mass spectrometry (MS) conditions were as follows: electrospray ion source (ESI) in positive and negative ionization mode; positive ion spray voltage, 3.50 kV; negative ion spray voltage, 2.50 kV; sheath gas, 30 arb; and auxiliary gas, 10 arb. The capillary temperature was 325 °C. The full scan was performed at a resolution of 60,000 in the range of 81-1000. Higher energy collision dissociation (HCD) was used for secondary cracking with a collision voltage of 30 eV. Dynamic exclusion was used to remove unnecessary MS/MS information.

### Statistical analysis

Data are expressed as the means ± standard deviations. Origin 2018 was used for graphing, and IBM SPSS Statistics 25 software was used for data analysis. One-way analysis of variance (ANOVA) followed by Duncan’s test was used for comparisons among groups. *P* < 0.05 [95% confidence interval (CI)] was considered statistically significant.

## Results and analysis

### Effects of HAS on the growth status of INS antagonist-treated mice

Compared with BG group, one week after STZ injection, the body mass of mice was significantly decreased (*P < 0.05*), (*P* < 0.05) and the typical DM symptoms such as polydipsia, polyphagia and polyuria appeared. Meanwhile, the activity level was decreased, the body hair was fluffy and dull, and the spirit was weak. Compared with the BG group, the MG group presented a significant (*P* < 0.05) 45.37% decrease in body weight, a significant (*P* < 0.05) 40.35% increase in food intake, and a significant (*P* < 0.05) 4.21-fold increase in water consumption. Compared with MG group, after treatment with different doses of HAS via gavage for 28 days, the body weights of the mice in each dose group tended to increase, in HG, DG and LG groups, with increases of 18.27%, 13.41% and 6.68%, respectively. Compared with those in the MG, the food intake and water consumption of the mice in each HAS DG were significantly (*P* < 0.05) lower, with food intake decreasing by 29.82%, 27.92% and 14.13% and water consumption decreasing by 53.82%, 50.93% and 51.76% in HG, DG and LG groups, respectively. However, food intake, water consumption and final body weight did not significantly differ between the PC group and the INS antagonist-treated group (*P* ≥ 0.05). These results indicate that HAS can relieve the symptoms of polydipsia, polyphagia and weight loss in mice with IR Table 1.

**Table 1:** Effect of HAS on the growth status of INS antagonist-treated mice (*n* = 12)

Group	Initial body weight (g)	Final body weight (g)	Food intake (g/w)	Water consumption (mL/w)
BG	54.30 ± 1.31^a^	56.26 ± 1.45^a^	33.82 ± 1.22^d^	40.50 ± 2.74^c^
MG	45.41 ± 1.36^b^	30.73 ± 1.41^d^	56.70 ± 1.89^a^	170.58 ± 5.43^a^
PC	48.59 ± 1.12^b^	41.42 ± 1.05^b^	38.61 ± 1.49^c^	79.48 ± 6.12^b^
HG	47.86 ± 1.56^b^	37.60 ± 1.44^bc^	39.79 ± 1.40^c^	78.77 ± 6.21^b^
DG	46.86 ± 1.02^b^	35.49 ± 1.51^c^	40.87 ± 1.28^c^	83.71 ± 6.09^b^
LG	47.27 ± 1.41^b^	32.93 ± 1.49^cd^	48.69 ± 1.59^b^	82.29 ± 8.21^b^

Note: Differences observed between samples are represented by lowercase letters (a, b, c) (*P* < 0.05)

### Effects of HAS on glucose metabolism indicators in INS antagonist-treated mice

**Fig. 1:**
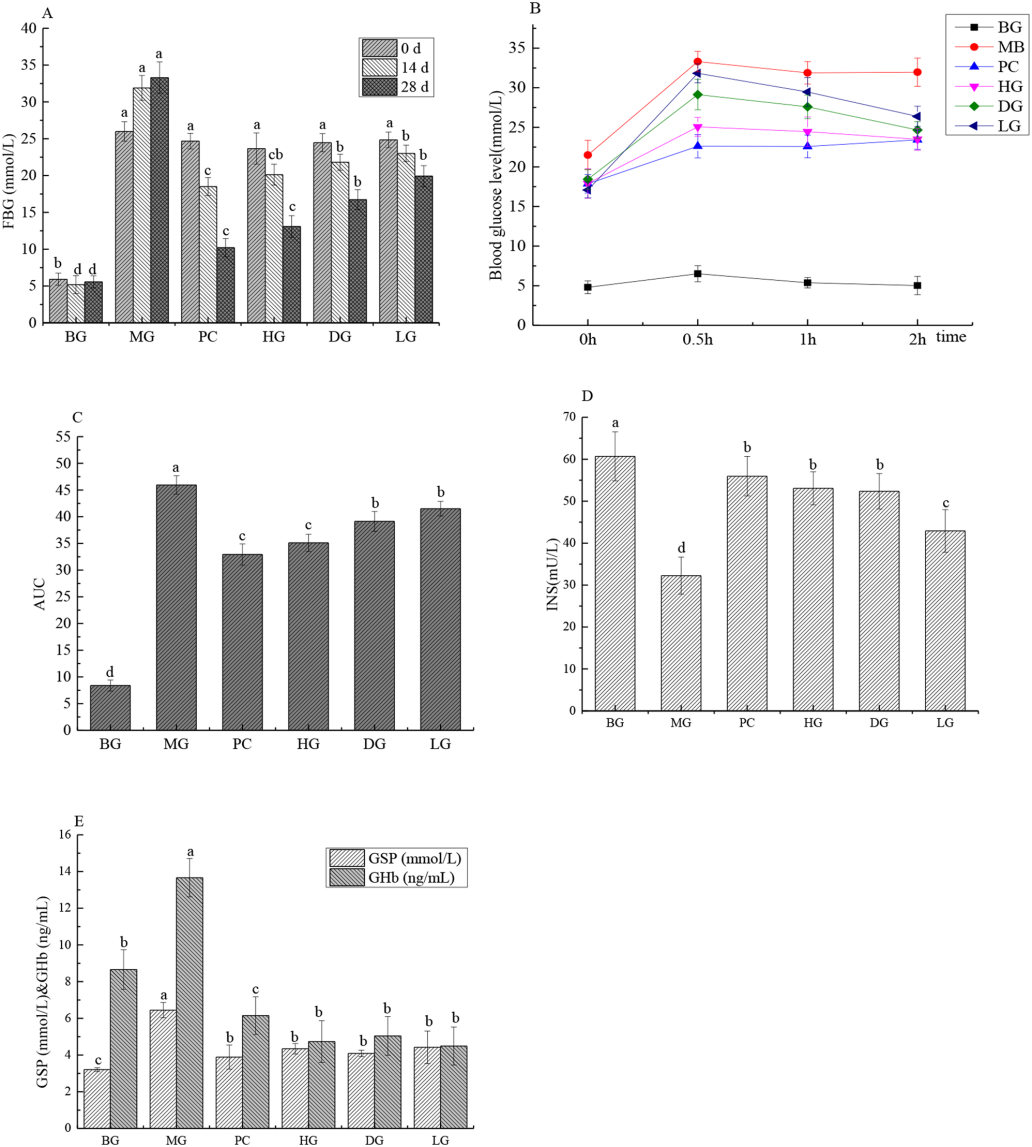
Effects of HAS on glucose metabolic indicators in INS antagonist-treated mice (*n* = 12) (**A**, FBG level; **B**, glucose tolerance test; **C**, Area under the curve (AUC); **D**, INS level; **E**, GSP and GHb levels)

FBG represents the basal blood glucose level in diabetic patients and reflects basal pancreatic islet function. As shown in Fig. 1, the FBG level of the mice in the BG was always within the normal range (4–6 mmol/L) during the feeding period, whereas that of the mice in the MG was greater than 11 mmol/L, indicating that the mouse model was successfully established and stable. Compared with those in the MG, after 2 weeks of HAS gavage, the FBG levels in the PC group, HG, DG and LG were 41.99%, 36.915%, 31.615% and 27.84% lower, respectively, than those before treatment; after 28 days of HAS gavage, the FBG levels in the PC group, HG, DG and LG were 69.34%, 60.69%, 49.76% and 40.18% lower, respectively (*P* < 0.05) (Fig. 1A). Oral glucose tolerance reflects the function of islet beta cells and the body’s ability to regulate glucose. The oral glucose tolerance curve of mice and the area under the curve (AUC) are shown in Fig. 1B and C. As can be seen from Fig. 1B, the glucose tolerance of the BC group was normal, while that of the MG group reached its highest value at 0.5 h, but remained at a high level thereafter, indicating impaired glucose tolerance. Compared with MG group, the blood glucose level of HAS and PC groups reached the highest value at 0.5 h, and then decreased at a faster rate, but failed to recover to the initial level, indicating that HAS could improve the glucose tolerance of insulin-resistant mice. Compared with BG group, the AUC value of MG group was significantly increased by 81.73% (*P* < 0.05), while the AUC value of MG group was significantly decreased by 23.60%, 14.84% and 9.67% after administration of different doses of HSA, respectively (*P* < 0.05). INS is the only substance in the body that causes hypoglycemia, and its secretion status and receptor function are related to DM (Skeldon et al. 2016). INS in the MG was significantly lower than that in the BG, suggesting that pancreatic islet cells were destroyed. GHb and GSP levels are positively proportional to the blood glucose concentration, and GHb and GSP levels in the MG were significantly greater than those in the DG (*P* < 0.05). After HAS intervention, the GHb and GSP levels in each group were significantly lower than those in the MG. These results suggest that HAS administration reduces FBG levels and promotes INS secretion in IR model mice, with significant effects on the HG and DG.

### Effects of HAS on serum lipid metabolism, oxidative stress and inflammatory cytokines in INS antagonist-treated mice

The results showed the effects of HAS on serum lipid metabolism, oxidative stress and inflammatory cytokines in INS antagonist-treated mice. As shown in Table 2, compared with those in the BG, the four lipid indicators in the MG changed significantly. TC, TG, and LDL-C levels significantly increased, whereas HDL-C levels significantly decreased (*P* < 0.05). Treatment with HAS decreased the serum TC, TG, and LDL-C levels and increased the HDL-C level. In the PC group, the HG, and the DG, serum TC, TG, and LDL-C levels were significantly lower, and the HDL-C level was significantly greater. Compared with those in the BG, the CAT, GSH-Px, and SOD levels were significantly lower in the MG, whereas the MDA level was significantly greater. Compared with those of the PC group, HAS significantly reduced the serum MDA level and significantly increased the CAT, GSH-Px, and SOD levels in the HG and DG (*P* < 0.05). Compared with those in the BG, the IL-1, IL-6, TNF-α, and MCP-1 levels in the MG were significantly greater, but the IL-2 level was significantly lower. Treatment with HAS reduced the serum IL-1, IL-6, TNF-α, and MCP-1 levels and increased the IL-2 level. The results revealed that HAS alleviated lipid metabolic disorders and reduced oxidative stress damage and inflammation in IR model mice, with significant effects on the HG and DG.

**Table 2:** Effects of HAS on serum lipid metabolism, oxidative stress and inflammatory cytokines in INS antagonist-treated mice (*n* = 12)

Group	TC (mmol/mL)	TG (mmol/mL)	HDL-C (mmol/L)	LDL-C (mmol/L)	CAT (U/mL)	GSH-Px (U/mL)	MDA (nmol/mL)	SOD (U/mL)	IL-1 (pg/mL)	IL-2 (pg/mL)	IL-6 (pg/mL)	TNF-α (pg/mL)	MCP-1 (pg/mL)
BG	3.08 ± 0.48^c^	1.18 ± 0.33^b^	3.43 ± 0.19^a^	0.22 ± 0.03^c^	156.43 ± 11.91^a^	360.35 ± 19.56^b^	35.25 ± 5.91^c^	223.65 ± 21.01^a^	435.23 ± 33.73^c^	513.89 ± 46.79^b^	194.86 ± 11.98^cb^	775.00 ± 59.75^d^	706.49 ± 25.96^c^
MG	7.06 ± 0.75^a^	2.53 ± 0.18^a^	1.87 ± 0.24^c^	0.58 ± 0.06^a^	94.45 ± 133.82^c^	229.97 ± 47.05^c^	76.11 ± 4.01^a^	156.21 ± 19.68^b^	737.61 ± 77.41^a^	321.39 ± 50.56^c^	280.67 ± 14.06^a^	1677.41 ± 24.57^a^	1302.39 ± 22.80^a^
PC	3.73 ± 0.57^bc^	1.51 ± 0.21^b^	3.29 ± 0.36^a^	0.30 ± 0.07^bc^	137.41 ± 11.48^a^	445.14 ± 53.21^a^	47.00 ± 7.22^bc^	215.35 ± 14.90^a^	584.21 ± 69.43^b^	839.31 ± 57.89^a^	231.75 ± 16.62^ab^	1089.028 ± 23.98^c^	769.14 ± 53.93^c^
HG	4.84 ± 0.43^b^	1.61 ± 0.27^b^	3.08 ± 0.45^a^	0.34 ± 0.05^b^	115.29 ± 9.94^b^	550.40 ± 73.19^a^	55.80 ± 9.27^b^	235.39 ± 12.52^a^	469.79 ± 50.96^c^	810.42 ± 29.05^a^	221.04 ± 15.67^ab^	1217.59 ± 28.63^b^	787.59 ± 58.43^c^
DG	5.57 ± 0.38^ab^	1.75 ± 0.24^b^	2.87 ± 0.49^ab^	0.46 ± 0.09^ab^	97.65 ± 12.07^bc^	395.23 ± 63.68^ab^	61.81 ± 5.21^b^	258.49 ± 22.65^a^	586.34 ± 56.06^b^	805.28 ± 51.93^a^	236.97 ± 10.86^ab^	1253.70 ± 49.21^b^	795.50 ± 44.77^c^
LG	6.00 ± 0.22^a^	2.04 ± 0.28^ab^	2.01 ± 0.43^bc^	0.56 ± 0.07^a^	97.80 ± 7.15^bc^	390.54 ± 80.26^ab^	65.66 ± 6.76^ab^	237.63 ± 18.68^a^	482.89 ± 21.12^c^	602.92 ± 50.99^b^	252.24 ± 15.54^a^	1304.72 ± 59.02^b^	913.34 ± 64.39^b^

Note: Differences observed between samples are represented by lowercase letters (a, b, c,) (*P* < 0.05), where “a, ab, bc, c” represent the variability between groups

### Effects of HAS on liver pathology in mice

**Fig. 2:**
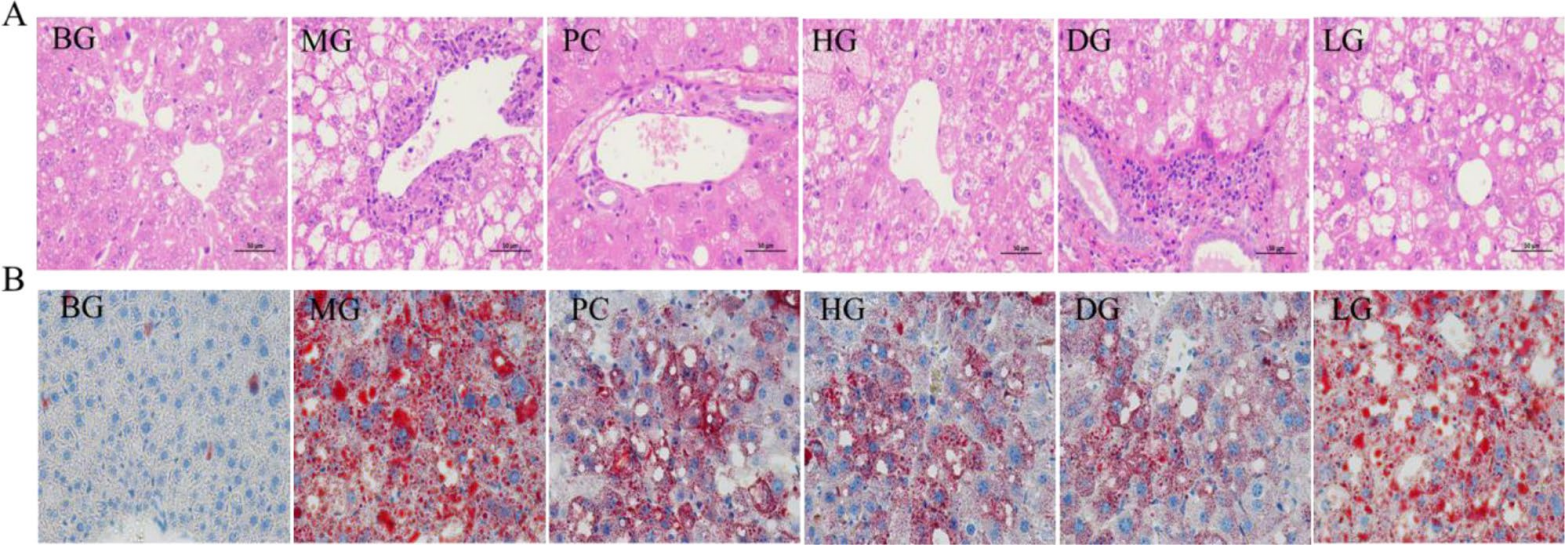
Effect of HAS on the pathological morphology of mouse liver tissues (×400). (**A**) HE staining, (**B**) Oil red O staining

As shown in Fig. 2A, the cells in the liver tissues of the mice in the BG were uniform in size, with a normal morphology, an abundant number of organelles, and a regular shape; additionally, the structure of the liver lobule was normal. In the MG, the tissue structure of the liver lobule was blurred, many fat vacuoles were observed between cells, the liver cells were swollen, the cell arrangement was disordered, and the structure of the liver cord was blurred. After treatment with HAS, the cell arrangement within the liver became significantly more ordered, and swelling, steatosis, and inflammatory cell infiltration decreased. Oil red O staining revealed severe lipid accumulation in the liver tissues of the mice in the MG, with lipid accumulation significantly decreasing in the liver tissue of the mice in the HAS groups after treatment (Fig. 2B). HE staining and oil red O staining revealed that the effects in the HG and DG were more significant than those in the other groups.

The physiological, biochemical and liver pathology results revealed that, in the HG and DG, the regulatory effects of HAS on the growth status, glycolipid metabolism, oxidative stress, inflammatory factors and liver pathology of the IR model mice were significant; however, the effects in the HG and DG were similar. Therefore, after comprehensively considering safety, economy, and omics data processing, we selected the DG for subsequent transcriptomic and metabolomic analyses.

### Transcriptomic analysis results

#### Volcano plot of DEGs and GO functional annotation

After sequencing quality control, the Q30% of each sample was no less than 91.48%, and the GC percentage was between 48.00% and 48.97%, indicating that the sequencing quality was good.

**Fig. 3:**
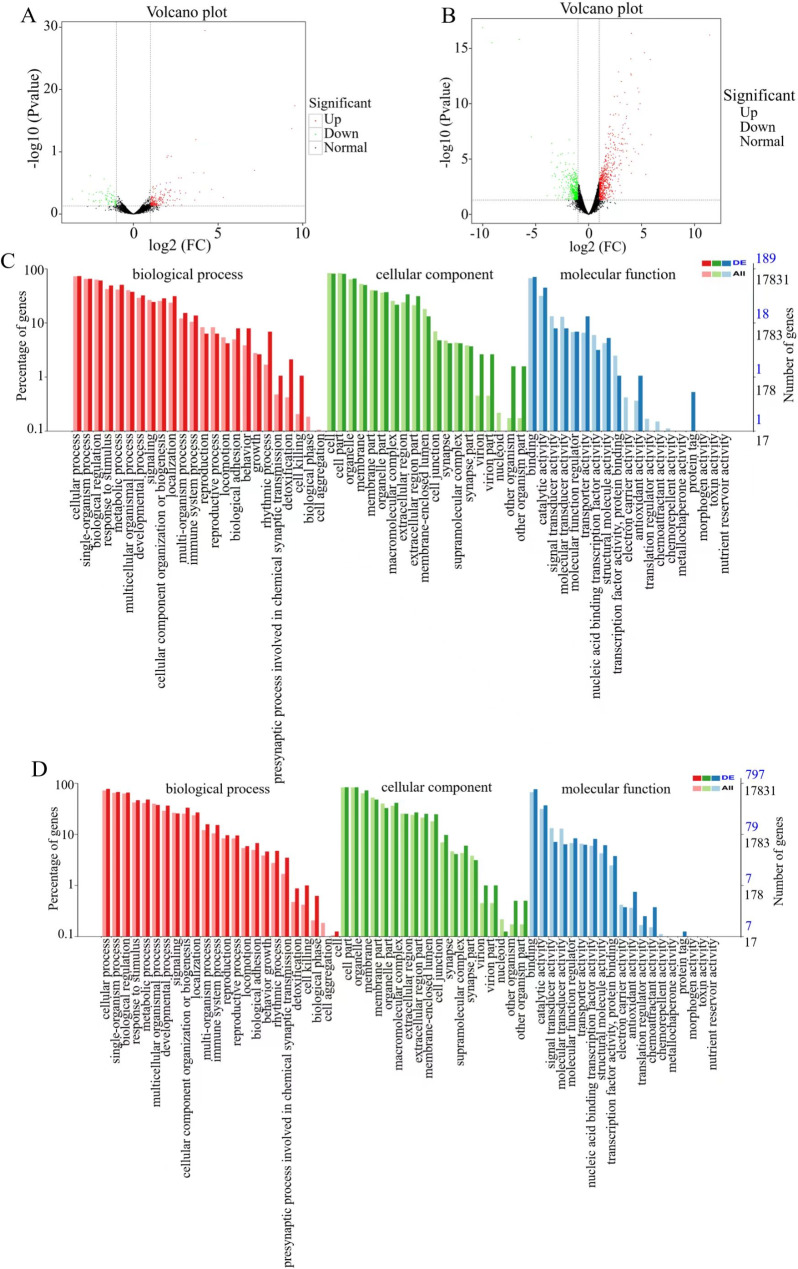
(**A**) Volcano plot of DEGs (BG vs. MG). (**B**) Volcano plot of DEGs (MG vs. DG). (**C**) GO functional annotation (BG vs. MG). (**D**) GO functional annotation (MG vs. DG)

The values of -log10 (*P* value) and log2 (FC) values were used to screen DEGs, with screening conditions of a *P* value less than 0.05 and a FC greater than 2. A volcano plot of the DEGs was generated. Each dot in the map represents a gene, the abscissa represents the logarithmic value of the FC in the gene expression between the two samples, and the ordinate indicates the negative logarithmic value of the statistical significance of the change in gene expression. The larger the absolute abscissa value was, the greater the FC in the expression level between the two samples; the larger the ordinate value was, the more significant the differential expression was, and the more reliable the DEGs obtained from the screening were. The volcano plot visually shows the up- and downregulation of the DEGs among the groups. As shown in Fig. 3A and B, when the BG was compared with the MG, the total number of DEGs was 252, with 183 upregulated genes and 69 downregulated genes; when the MG was compared with the DG, the total number of DEGs was 1083, with 610 upregulated genes and 473 downregulated genes.

After significant DEGs were screened, GO functional analysis was performed. The abscissa represents the GO classification, the left side of the ordinate represents the percentage of the number of genes, and the right side represents the number of genes. GO is divided into three components: biological process (BP), cellular component (CC) and molecular function (MF). As shown in Fig. 3C, for BPs, the DEGs of the BG vs. the MG were involved mainly in cellular processes (139), single-organism processes (124), biological regulation (115), metabolic processes (96), and response to stimuli (93); for CCs, the DEGs of the BG vs. the MG were involved mainly involved in the cell (154), cell parts (153), organelles (125), membranes (95), and membrane parts (75); and for MFs, the DEGs of the BG vs. the MG were involved mainly in binding (134), catalytic activity (85), transporter activity (25), molecular transducer activity (15), and signal transducer activity (15). As shown in Fig. 3D, for BPs, the DEGs of the MG vs. the DG were involved mainly in cellular processes (621), single-organism processes (541), biological regulation (527), metabolic processes (384), and response to stimuli (372); for CCs, the DEGs of the MG vs. the DG were involved mainly in the cell (669), cell parts (667), organelles (580), membranes (384), and organelle parts (332); and for MFs, the DEGs of the MG vs. the DG were involved mainly in binding (612), catalytic activity (296), molecular function regulation (67), nuclear acid binding transcription factor activity (65), and signal transducer activity (57).

#### KEGG pathway enrichment analysis

**Fig. 4:**
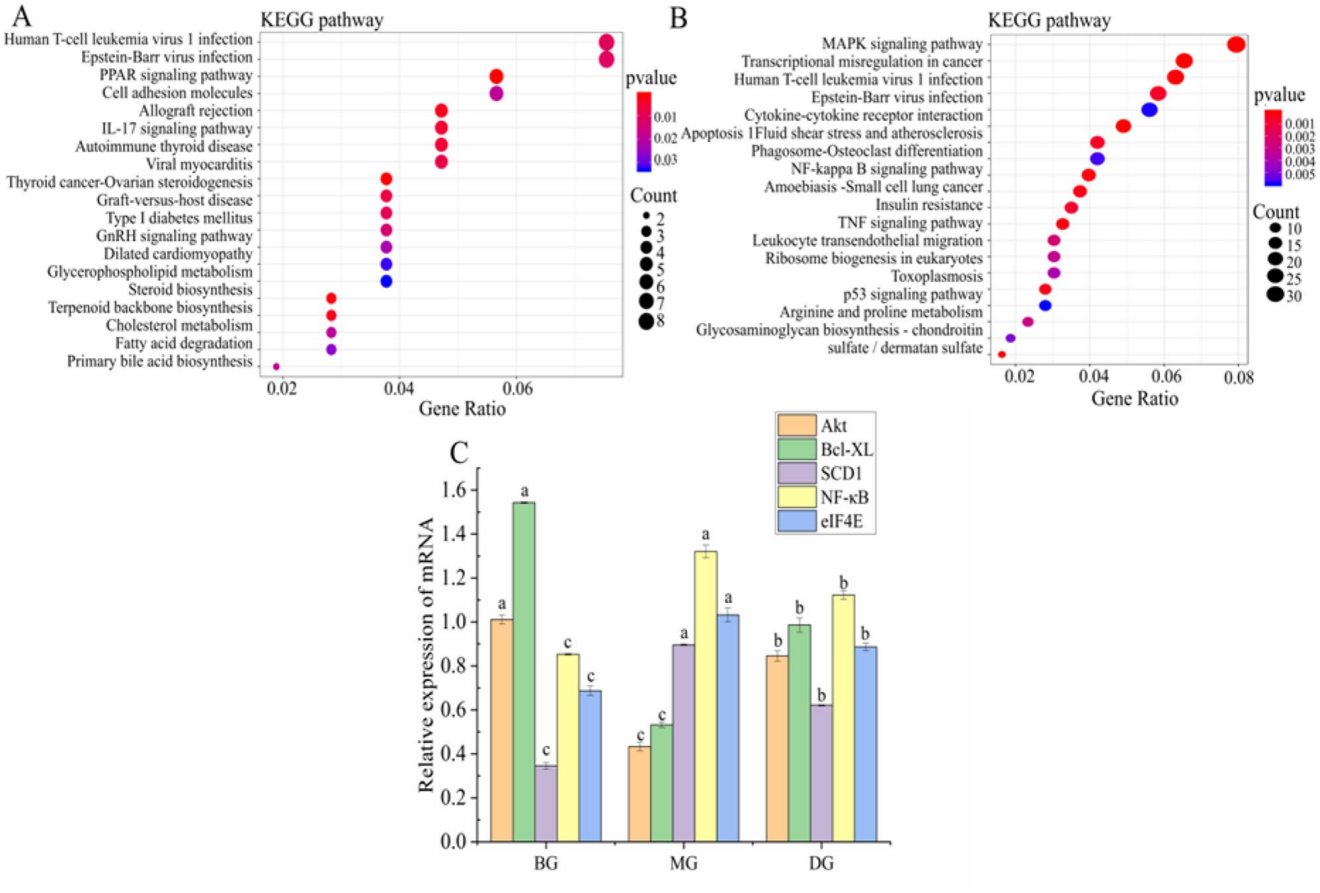
(**A**) Bubble chart of the KEGG enrichment analysis of DEGs (BG vs. MD). (**B**) Bubble chart of the KEGG enrichment analysis of DEGs (MG vs. DG). (**C**) qRT‒PCR validation results

To further elucidate the functions of the DEGs, we performed KEGG pathway enrichment analysis on the DEGs. Pathway enrichment can identify the main biochemical metabolic pathways and signal transduction pathways associated with DEGs. Pathway enrichment analysis uses the pathways in the KEGG database as the unit and applies a hypergeometric test to identify pathways that are significantly enriched in DEGs compared with the whole-genome background. The genes were ranked by gene ratio, with *P* < 0.05 as the threshold of significant enrichment. The results (Fig. 4A and B) revealed that the 252 DEGs in the BG vs. the MG were enriched mainly in the following pathways: the PPAR signaling pathway, the IL-7 signaling pathway, and fatty acid degradation. Compared with those in the MG, the 1083 HAS-induced DEGs were clustered into the MAPK signaling pathway, NF-kappa B signaling pathway, TNF signaling pathway, IR pathway, and P53 signaling pathway. The statistics of the significant genes associated with the enriched KEGG pathways in Fig. 4 are presented in Supplementary Table S2, and the genes most strongly correlated with IR were Akt, B-cell lymphoma extra-large (Bcl-xL), SCD1, nuclear factor kappa B (NF-κB), and eukaryotic translation initiation factor 4E (eIF4E). HAS reversed the decreased Akt and Bcl-xL levels in the MG and increased the expression levels of these genes. HAS also reversed the increased SCD1, NF-κB, and eIF4E levels and decreased the expression levels of these genes, suggesting that the mechanism by which HAS alleviates IR is associated with the upregulation of Akt and Bcl-xL gene expression and the downregulation of SCD1, NF-κB, and eIF4E gene expression.

#### qRT‒PCR results

The key genes identified via transcriptome screening were validated via qRT‒PCR, and the results are shown in Fig. 4C. Compared with those in the BG, the mRNA expression levels of Akt and Bcl-xL in the MG were significantly lower by 57.17% and 65.52%, respectively, whereas the mRNA expression levels of SCD1, NF-κB and eIF4E in the MG were significantly greater by 61.50%, 35.38% and 33.43%, respectively. After treatment with HAS, the mRNA expression levels of Akt and Bcl-xL significantly increased by 48.78% and 46.07%, respectively, whereas the mRNA expression levels of SCD1, NF-κB and eIF4E significantly decreased by 30.69%, 15.00% and 14.10%, respectively. These results were consistent with the RNA-seq analysis results.

### Serum metabolomic analysis of mice with IR

To further explore the ability of HAS to alleviate IR in mice, we analyzed mouse sera via metabolomic technology. A total of 8417 precursor molecules were detected in positive ion mode, and a total of 6973 precursor molecules were detected in negative ion mode. Differentially abundant metabolites were screened by combining a *P* value ≤ 0.05 and VIP ≥ 1 with a one-way ANOVA *P* value ≤ 0.05 and VIP ≥ 1. There were 354 differentially abundant metabolites identified in positive ion mode and 278 differentially abundant metabolites identified in negative ion mode.

**Fig. 5:**
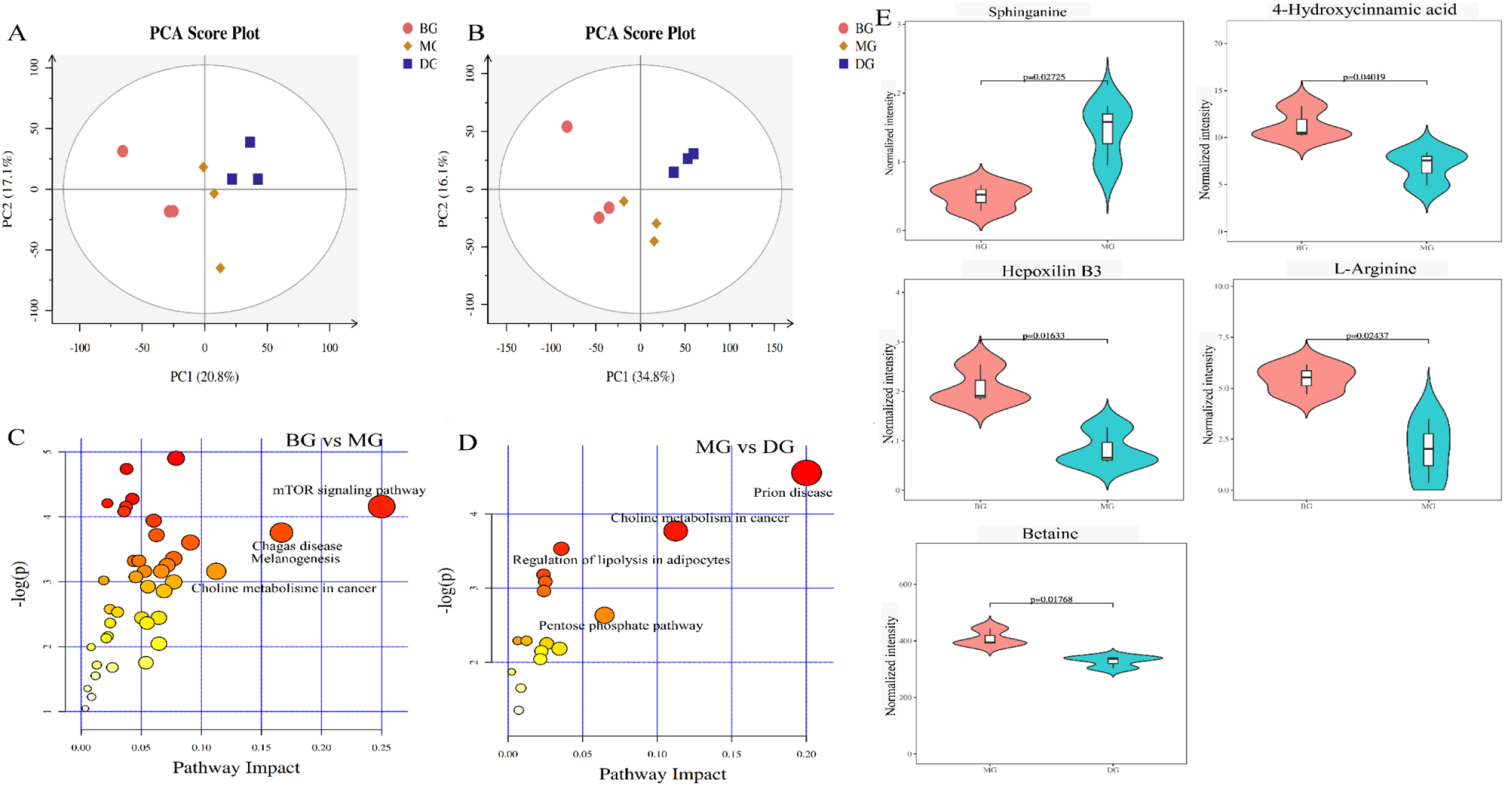
(**A**) (**B**) PCA results for mouse serum metabolomics. (**A**) Positive ion mode; (**B**) negative ion mode. (**C**) (**D**) Bubble chart of factors influencing metabolic pathways. The redder and larger the bubbles are, the greater the impact on the pathway. (**E**) Abundance of differentially abundant metabolites between samples

Statistical analysis was performed on the differentially abundant metabolites, and the principal component analysis (PCA) results are shown in Fig. 5A, B. In positive ion mode (Fig. 5A), the intersample model interpretability R^2^X = 0.532, PC1 = 20.8%, and PC2 = 17.1%. In negative ion mode (Fig. 5B), the model interpretability was R^2^X = 0.509, PC1 = 34.8%, and PC2 = 16.1%. In general, an R^2^ higher than 0.5 indicates that a model is good. The PCA plot revealed that the BG, MG and HG samples presented a high degree of intragroup aggregation and high intergroup dispersion, indicating that there were differences among the samples and that the data were independent and reliable. Therefore, an in-depth analysis of differentially abundant metabolites could be performed.

Figure 4D presents a bubble chart of the metabolic pathway influencing factors. Compared with those in the BG, the differentially abundant metabolites in the MG were enriched mainly in the mTOR signaling pathway, Chagas disease, melanogenesis, and choline metabolism in cancer (Fig. 5C). After treatment with HAS, the differentially abundant metabolites in the DG affected mainly prion disease, choline metabolism in cancer, the pentose phosphate pathway, and the regulation of lipolysis in the adipocyte signaling pathway (Fig. 5D). In summary, HAS alleviated IR in mice by affecting physiological metabolism, oxidative stress, and glucose catabolism.

The relevant differentially abundant metabolites among the three samples were screened on the basis of mouse pathology (Fig. 5E). Compared with those in the BG, the differentially abundant metabolites in the MG included sphinganine, 4-hydroxycinnamic acid (HA), hepoxilin B3, and L-arginine; the abundance of sphinganine was increased, whereas the abundances of HA, hepoxilin B3, and L-arginine were decreased. Sphinganine and sphinganine kinase 2 in the liver are key regulators of glucose homeostasis and INS sensitivity, whereas sphinganine is an endogenous inhibitor of INS signal transduction in the liver. High sphinganine levels can promote IR in sphinganine kinase 2-deficient hepatocytes (Aji et al. 2020). HA can offset the inhibitory effect of dorsomorphin (a selective AMPK inhibitor) on AMPK activation and can also regulate glucose and lipid metabolism in the body in combination with AMPK agonists to prevent or alleviate IR (Guan et al. 2018). Hepoxilin B3 is a 12-lipoxygenase metabolite of arachidonic acid and an INS release enhancer in mammals (Moghaddam et al. 1990a, b). L-arginine plays an important role in glucose metabolism. L-arginine can induce INS secretion, reduce obesity, reduce plasma glucose levels, alleviate INS sensitivity and alleviate IR (Forzano et al. 2023). The expression levels of Hepoxilin B3 and L-arginine decreased in the MG, indicating that the INS level decreased and that IR developed. After treatment with HAS, the abundance of the differentially abundant metabolite betaine decreased. Betaine is involved in lipid metabolism and inflammatory inhibition through fatty acid oxidation and phosphatidylcholine synthesis (Wray-Cahen et al. 2004). FOXO transcription factors are regulated by INS signal transduction. The NLRP3 inflammasome can be activated by IR and FOXO1, resulting in increased expression of the inflammatory factor IL-1β. Moreover, betaine can regulate the association between FOXO1 and NLRP3 at the cellular and molecular levels and is a newly identified target for the treatment of DM (Kim et al. 2017). In summary, the intake of HAS positively regulates the abundance of betaine, indicating that HAS indirectly alleviates high-fat-induced glucose metabolic disorders and IR.

### Correlation analysis of inflammatory factors with DEGs and differentially abundant metabolites in mice with IR

To further elucidate the potential links between inflammatory factors and key differentially abundant metabolites and DEGs, we constructed a heatmap based on Spearman correlation analysis, as shown in Fig. 7A and B. We found that the inflammatory factors IL-1, TNF-α, IL-6, and MCP-1 were significantly positively correlated with the differentially abundant metabolite sphinganine and significantly negatively correlated with L-arginine and Hepoxilin B3. IL-1, TNF-α, IL-6, and MCP-1 were significantly positively correlated with the DEGs NF-κB, SCD1, and eIF4E, whereas they were significantly negatively correlated with Akt and Bcl-xL.

## Discussion

The results of the present study demonstrated that an appropriate amount of HAS can significantly reduce the serum GSP and GHb levels; significantly increase the INS level in IR model mice; significantly alleviate lipid metabolic disorders in the serum, liver and kidneys; reduce oxidative stress damage in the serum, liver and kidneys of IR model mice; and significantly reduce the levels of inflammatory factors, such as IL-1, IL-6, TNF-α, and MCP-1, in the serum and liver of IR model mice. The pathological results revealed that HAS alleviated hepatic steatosis and inflammation in mice with IR. Long-term high-fat diets cause elevated lipids, with specific results in the form of increased TC, TG, and LDL-C contents and decreased HDL-C contents. Such abnormalities in lipid metabolism can exacerbate the degree of IR (Chen et al. 2019). Xu et al. (2022a, b) showed that the intake of HAS can effectively regulate lipid content and alleviate abnormalities in lipid metabolism, which is consistent with the findings of this study.

CAT, GSH-Px, SOD and MDA are important indicators for evaluating the antioxidant capacity of the body. Oxidative stress can exacerbate pancreatic β-cell damage and affect pancreatic islet function, which in turn elevates blood glucose. Elevated blood glucose in turn exacerbates oxidative stress and induces apoptosis, which ultimately leads to a vicious cycle (Sun et al. 2015). One group reported that HAS can protect the activity of antioxidant enzymes and ameliorate oxidative stress by increasing the levels of CAT and GSH-Px and decreasing the levels of MDA in different disease models (Li et al. 2024a, b). This study also revealed that HAS is an effective antioxidant that ameliorates oxidative stress in mice with IR by scavenging oxidizing substances and increasing antioxidant enzyme activities. As reported in the literature, elevated glucose levels have been shown to induce the synthesis of IL-6 and TNF-α by single nucleated cells (Jones et al. 2021). TNF-α, a multifaceted cytokine, is present at very low levels in most normal organisms and increases rapidly when exposed to inflammatory factors (Umapathy et al. 2018). MCP-1 expression is induced by TNF-α. IL-6, a pivotal component of the cytokine network induced by IL-1 and TNF-a, has been correlated with the progression of inflammation (Karakas et al. 2022). IL-2 is secreted primarily by activated T cells, whereas mice in the MG group exhibited reduced IL-2 secretion due to impaired autogenous immune system function, and IL-2 levels increased after HAS intervention, suggesting that HAS can promote immunity. In a previous study, the intake of HAS promoted INS secretion, and with the subsequent alleviation of IR, the levels of the inflammatory factors IL-1, IL-6, TNF-α, MCP-1, and IL-2 were significantly restored. HAS has been shown to act as an insulin sensitizer, inhibiting the expression of certain inflammatory genes through the activation of PPAR-γ and regulating the production of inflammatory cytokines by monocytes/macrophages and the release of inflammatory mediators.

**Fig. 6:**
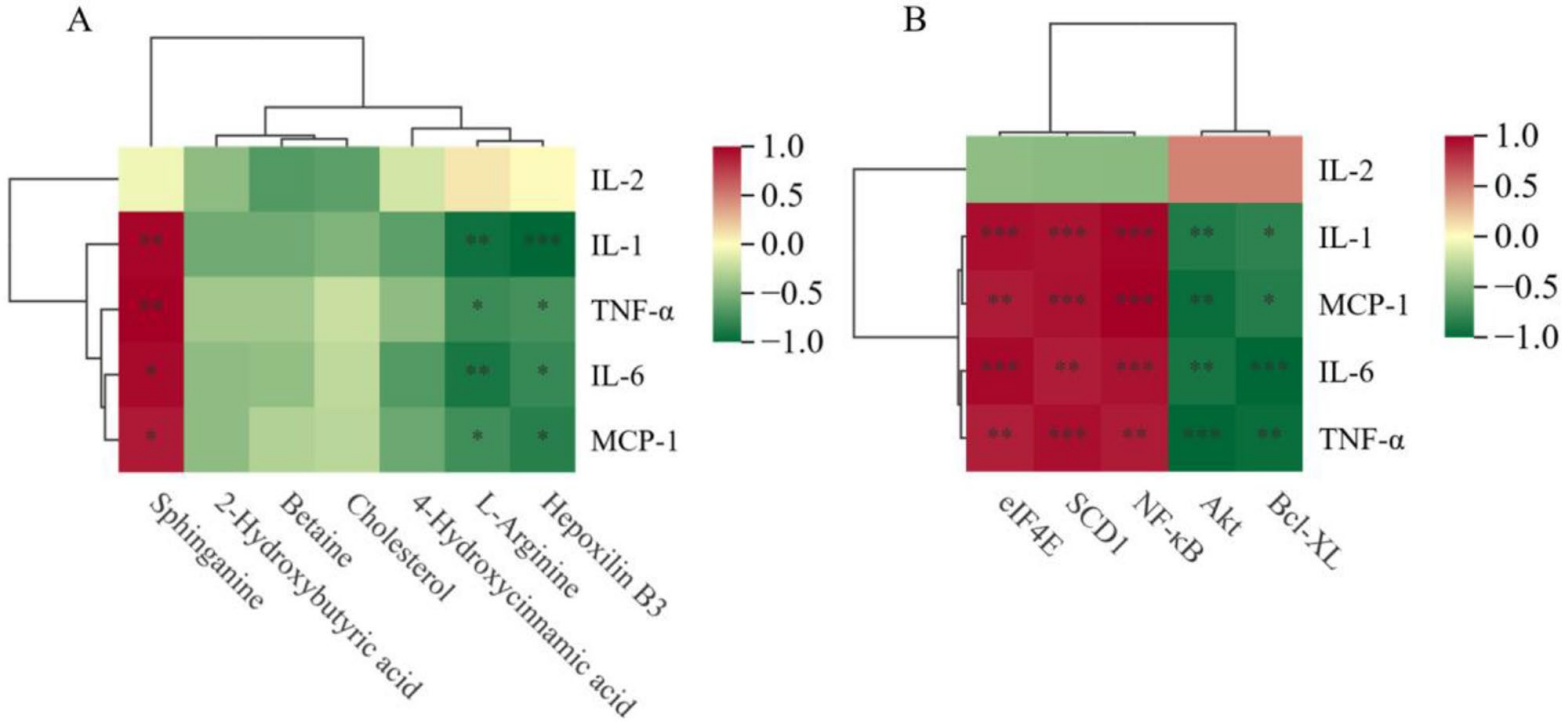
Spearman correlation analysis of differentially abundant metabolites-inflammatory factors-DEGs. ***P < 0.001, **P < 0.01, *P < 0.05. (**A**) Correlation analysis of inflammatory factors and differentially abundant metabolites. (**B**) Correlation analysis of inflammatory factors and DEGs (red and green indicate positive and negative correlations, respectively)

The effect of HAS on IR was related to the activation of the PI3K/Akt INS signaling pathway and the NF-kB signaling pathway (Fig. 6). HA can promote the phosphorylation of AMPK (Yuan et al. 2023) and significantly increase glucose consumption in cells. Numerous studies have shown that liver-specific AMPK inhibition is associated with hepatic steatosis and inflammation. In addition, AMPK phosphorylation can inhibit lipogenesis and normalize glucose metabolism through the phosphorylation or inactivation of acetyl-CoA carboxylase (ACC) (Goodarzi et al. 2023). In addition, HAS can attenuate NF-κB-related inflammasomes, thereby attenuating inflammasome-related liver signaling pathway activation. NF-κB is a transcription factor that plays an important role in the regulation of inflammation (Lumeng et al. 2007) and is associated with IR and abnormal pancreatic β-cell function in metabolic syndrome. The pharmacological activation of NF-κB-inducible kinase (NIK) disrupts glucose homeostasis in zebrafish, indicating that NIK is a key negative regulator of β-cell function (Malle et al. 2015), a result that is consistent with the findings of this study. A variety of molecular mechanisms are closely related to the prevention and alleviation of various liver diseases and related complications. By increasing AMPK activity and reducing hepatic lipogenesis, betaine can reduce hepatic steatosis. In addition, betaine can attenuate IR. Vesković et al. reported that betaine significantly reduced the activities of alanine aminotransferase (ALT) and aspartate aminotransferase (AST) and the levels of LDL-C, ROS, and nitric oxide (NO) and increased the HDL-C level to reduce hepatic steatosis (Veskovic et al. 2019). In addition, the effects of betaine on liver fat are related to a reduction in inflammation, an increase in the number of mitochondria in apoptotic liver cells, an increase in cytoprotective Akt/mTOR signaling, and an increase in the number of autophagosomes (Vesković et al. 2020). In addition, signaling pathways, such as the NF-κB, AMPK, and Akt/mTOR pathways, are considered important molecular targets for the alleviation of various liver diseases by betaine (Wang et al. 2021), and these results are consistent with the findings of our study. Sphingolipid metabolites, including sphinganine and other long-chain bases, can effectively induce apoptosis in human liver cancer cells. Akt [also known as protein kinase B (PKB)] is a serine/threonine kinase that induces the transport of the glucose transporter protein GLUT to the cell membrane, thereby increasing glucose uptake (Sharma et al. 2021).

Akt is located almost at the center of the INS signaling cascade and is the main universal node of INS signal transduction, and a deficiency in Akt activity triggers IR (Toker and Marmiroli 2014). Akt has isoforms that endow it with multiple functions. Akt2 knockout mice exhibited a diabetic phenotype accompanied by hyperglycemia, impaired INS action, reduced circulating leptin, and mild growth retardation (George et al. 2004), whereas Akt2 and Akt3 double knockout mice exhibited hyperinsulinemia, hyperglycemia, and reduced brain volume (Dummler et al. 2006). As a downstream regulatory protein of the Akt pathway, Bcl-xL can form a complex with the key autophagy-related protein Beclin1 to regulate pancreatic islet cell apoptosis and normalize glucose metabolism (Chen et al. 2009; Tomita 2016). Mice with targeted destruction of the SCD1 locus are thin and exhibit increased energy consumption and INS sensitivity, and reducing SCD1 expression is a biochemical measure to alleviate INS sensitivity and glucose tolerance (Cohen et al. 2002). Oxidative stress can cause IR by promoting the expression of proinflammatory cytokines, such as TNF-α and IL-6, activating JNK, or causing oxidative damage to important macromolecules in INS-sensitive tissues, whereas NF-κB not only regulates TNF-α and IL-6 expression but also increases ROS and oxidative stress (Evans et al. 2003; Ogihara et al. 2004). Therefore, NF-κB is a key factor in the pathogenesis of IR caused by oxidative stress. eIF4E inhibits translation by binding to eIF4E-binding proteins (4E-BPs), and 4E-BP1 and 4E-BP2 double-knockout mice exhibited increased IR and impaired Akt signaling (Pause et al. 1994; Bacquer et al. 2007). In summary, HAS can maintain glucose homeostasis and regulate IR by regulating the expression levels of Akt, Bcl-xL, SCD1, NF-κB and eIF4E.

INS is an effective stimulator of Akt kinase, and the inhibition of Akt kinase is one of the mechanisms of sphinganine-induced apoptosis in liver cancer cells. Akt activation inhibits sphinganine-induced cell apoptosis possibly by blocking the upstream steps of cytochrome C release and caspase-3 activation (Chang et al. 2001). The PI3K/Akt signaling pathway is believed to play a key role in INS signal transduction and glucose metabolism regulation. INS usually acts by binding to its cell surface receptor (INSR), which activates the intrinsic tyrosine kinase activity of INSR, resulting in the autophosphorylation of INSR and the phosphorylation of several substrates. Phosphorylated INSR can bind to IRS-1 to transmit INS signals. IRS-1 subsequently activates Akt phosphorylation through PI3K (Wu et al. 2020). Zundel suggested that ceramide may directly inhibit PI3K activity to block Akt activation (Zundel and Giaccia 1998). In contrast, Zhou et al. reported that the effect of ceramide on Akt kinase was not mediated through the regulation of PI3K activity (Zhou et al. 1998). Whether sphinganine can directly affect PI3K or Akt activity is still unclear and is an important issue for future research. Hepoxilin B3 is a multifunctional cyclic hydroxy fatty acid that has been confirmed to have INS-secretagogue activity (Moghaddam et al. 1990a, b). L-arginine (Arg) is an important amino acid in nutrition, health and disease, and L-Arg metabolic pathways produce NO, polyamines, proline, glutamate, creatine and agmatine. NO plays a key role in physiological functions, such as the regulation of glucose and fatty acid metabolism (Alam et al. 2013). Since amino acids are precursors of the key cellular signaling molecule NO, the role of Arg is limited. Previous studies have indicated that the small amount of NO produced by NF-κB-regulated iNOS is an important coupling factor in INS-secreting cells. In rats fed a high-fat diet, the addition of Arg can reduce the serum glucose concentration (Jobgen et al. 2009). The activation of AMPK by Arg can promote fatty acid oxidation and improve the functional integrity of β-cells and pancreatic islets (Krause et al. 2011). AMPK negatively regulates INS secretion and impairs β-cell metabolism through the inhibition of mTOR, resulting in a decrease in INS secretion (Tuo et al. 2019). Although HAS may regulate IR through the activation of PI3K/Akt, the INS signaling pathway and NF-kB, more in-depth mechanistic studies, such as specific gene knockout or overexpression experiments, are needed to clarify the molecular mechanism of HAS further.

NF-κB induces the expression of various proinflammatory factors and is also involved in inflammatory regulation (Liu et al. 2017). SCD1 saturated fatty acids (SFAs) desaturate to produce monounsaturated fatty acids (MUFAs). The increase in the expression of proinflammatory markers is significantly ameliorated by the knockdown of SCD1 (Lounis et al. 2016). Similarly, eIF4E mediates inflammatory responses (Mody et al. 2020). Butyric acid inhibits inflammation and induces apoptosis by upregulating mTOR through the Akt signaling pathway (Pei et al. 2021). Bcl-xL may play an important role in the remission phase of acute inflammatory responses in vivo by affecting apoptosis (Sawatzky et al. 2006). The positive correlation between sphinganine and IL-6 reported by Wang et al. confirms the activation of inflammation by sphinganine (Wang et al. 2023). L-Arg, a basic half-amino acid, attenuates the inflammatory response by downregulating the expression of inflammatory factors (Kim et al. 2023). These results indicated that HAS ameliorated the inflammatory response in mice with IR by regulating the expression of key genes and metabolites.

Although HAS may regulate IR through the activation of the PI3K/Akt, INS signaling and NF-kB signaling pathways, more in-depth mechanistic studies, such as specific gene knockout or overexpression experiments, are needed to clarify the molecular mechanism of HAS further.

**Fig. 7:**
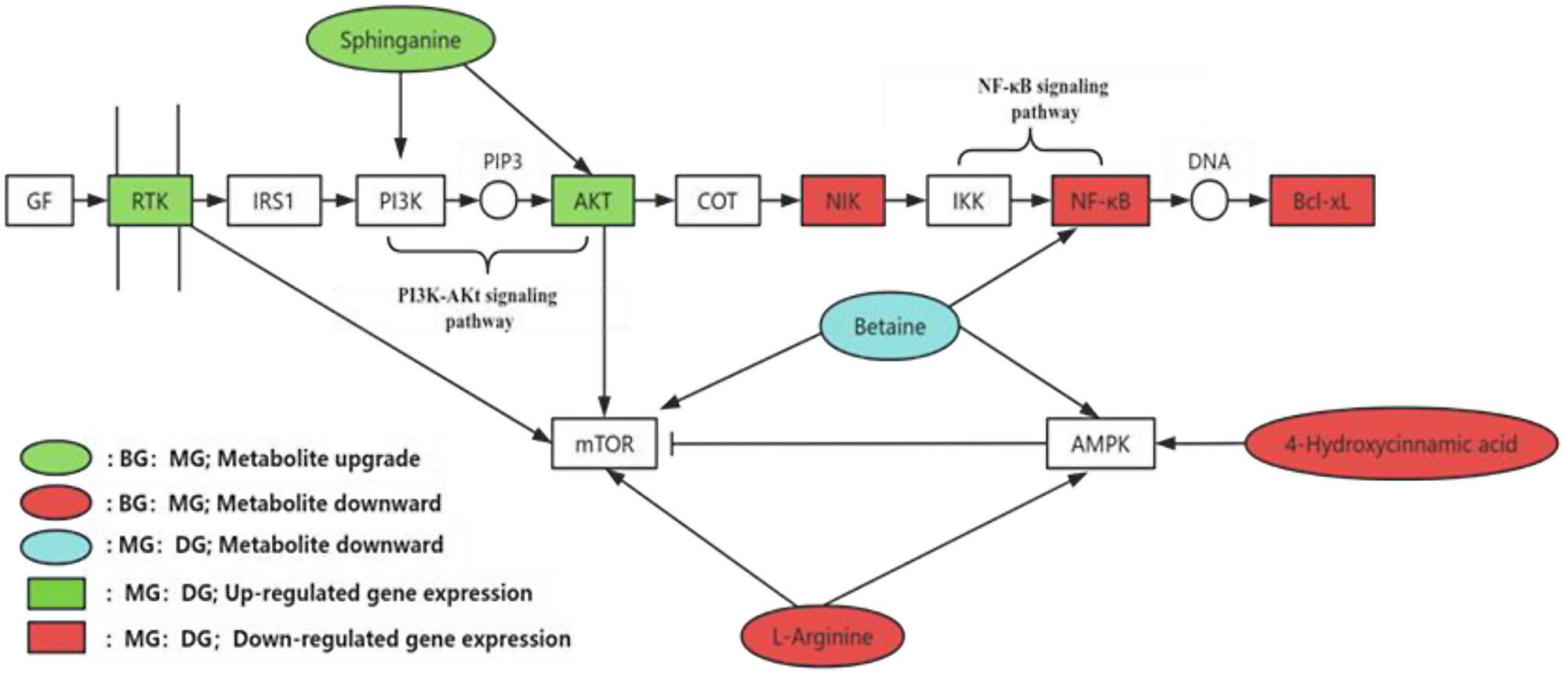
Comprehensive analysis of correlations among the DEGs and differentially abundant metabolites

## Conclusion

The findings of the present study demonstrated that moderate amounts of HAS could significantly reduce serum GSP and GHb levels and increase INS levels in IR model mice, thereby helping to alleviate lipid metabolic disorders, oxidative stress injury, and inflammation. These findings suggest that HAS exerts a preventive effect against hepatic vacuolating steatosis in IR model mice and that the underlying mechanism may be associated with the activation of the PI3K/Akt/INS signaling pathway and the NF-kB signaling pathway.

## Electronic supplementary material

Below is the link to the electronic supplementary material.

Supplementary Material 1

Supplementary Material 2

Supplementary Material 3

## Data Availability

No datasets were generated or analysed during the current study.
