# Supplementary material for: Integrated metabolomics and transcriptomics reveal the potential of hydroxy-alpha-sanshool in alleviating insulin resistance
Source: Mol Med. 2025 Feb 21;31:76. doi: 10.1186/s10020-025-01129-1 (PMC11846303; doi:10.1186/s10020-025-01129-1)
Supplement: Supplementary file 3 — Supplementary Material 3 [file 10020_2025_1129_MOESM3_ESM.docx]

**Supplementary materials**

Table S1 Primer sequences for qRT‒PCR

| Gene name | upstream primer (5'-3') | downstream primer (5'-3') |
| --- | --- | --- |
| Akt | CTCATTCCAGACCCACGAC | ACAGCCCGAAGTCCGTTA |
| Bcl-xL | CGTGGAAUGCGTAGACAGG | AAGGTGAGCCCAGCAGAAC |
| SCD1 | GGACTTCCTTCATTGCCAACA | CGCACCAGCCAACCCACACCAG |
| NF-κB | TGCCTCAGATACCTCACTCA | CAGCTTCTAGTTGTAGCTCGT |
| eLF4E | GACACTGCTGTCCTTATTG | CTTCGGAGGAGTCCTAUCC |
| β-actin | TGCTGTCCCTGTATGCCTCT | TTTGATGTCACGCACGATTT |

Table S2 Core enriched genes in KEGG pathways

| Pathway | Genes |
| --- | --- |
| Epstein‒Barr virus infection | AKT, Bcl-XL, and NF-κB |
| TNF signaling pathway | AKT, Bcl-XL, and NF-κB |
| NF-kappa B signaling pathway | Bcl-XL and NF-κB |
| PI3K-Akt signaling pathway | AKT, Bcl-XL, and NF-κB |
| MAPK signaling pathway | AKT and NF-κB |
| Insulin resistance | AKT and NF-κB |
| PPAR signaling pathway | SCD1 |
| RNA transport | eIF4E |
| Fatty acid degradation | SCD1 |
